# Invited Review: Inflammation and Health in the Transition Period Influence Reproductive Function in Dairy Cows

**DOI:** 10.3390/ani15050633

**Published:** 2025-02-21

**Authors:** Tony C. Bruinjé, Stephen J. LeBlanc

**Affiliations:** 1 Department of Dairy and Food Science, South Dakota State University, Brookings, SD 57007, USA; 2 Department of Population Medicine, University of Guelph, Guelph, ON N1G 2W1, Canada; sleblanc@uoguelph.ca

**Keywords:** disease, fertility, metabolism, peripartum, postpartum

## Abstract

In the early postpartum period, dairy cows undergo significant adaptations in Ca and lipid metabolism, immune function, and inflammatory processes. Concurrent exposure to endotoxins from the uterus, gastrointestinal tract, or mammary gland increases the risk of disease and reproductive problems. Metabolic and inflammatory imbalances during this phase can have both immediate and long-term effects on reproductive health. Associations between metabolic disorders and reproductive outcomes are often confounded by immune activation and systemic inflammation. However, optimal markers, thresholds, and durations for identifying maladaptation and predicting adverse health or reproductive outcomes remain unclear. This narrative review examines key physiological changes during the transition period, including hypocalcemia, lipid mobilization, immune activation, systemic inflammation, and uterine disease. We discuss how these events may affect the dominant follicle, corpus luteum, oocyte, and uterus, potentially leading to prolonged anovulation, reduced estrus expression, impaired response to synchronization protocols, lower progesterone concentrations, and compromised fertility. Understanding these mechanisms will support the development of strategies to monitor, prevent, and mitigate the impacts of transition-related maladaptation on reproductive performance. Such advancements can enhance the health and fertility of high-producing dairy cows.

## Simple Summary

During the period immediately after calving, dairy cows undergo critical physiological changes as they adapt to the postpartum period and the initiation of lactation. This phase involves crucial adjustments in metabolic and immune functions that can lead to health and fertility issues. Our review focuses on how immune dysfunction, uterine and systemic inflammation, and metabolic imbalance such as hypocalcemia and excessive lipid mobilization can influence reproductive processes of postpartum dairy cows, and result in decreased reproductive performance. By enhancing the understanding of these physiological changes, strategies that better support the health and fertility of dairy cows can be developed and implemented. This, in turn, will lead to healthier cows and to a more efficient and sustainable dairy production.

## 1. Introduction

Over the past decades, the dairy industry has undergone dramatic changes and substantial advancements in genetic selection, nutritional management, and herd management practices, resulting in averages milk production per cow per year of approximately 10,800 kg in the US and 11,100 kg in Canada [1,2]. To support milk production, the onset of lactation is characterized by drastic physiological and metabolic changes that start before parturition. Some of these challenges include immune activation, energy, protein and mineral imbalance, and uterine and systemic inflammation [3,4,5]. Adaptations related to Ca and lipid metabolism, immune function, and inflammation often occur concurrently in the early postpartum period when cows are exposed to endotoxins from one or more of the uterus, gut, or mammary glands, increasing the risk of developing disease and reproductive disorders.

Peripartum immune activation and changes in markers of systemic inflammation start at least 3 weeks before calving, which may be partially attributed to alterations in gastrointestinal tract permeability due to dietary changes, adipose tissue mobilization, mammary gland tissue expansion, and uterine tissue trauma during parturition [4,6]. Although uterine tissue trauma and inflammation during parturition are parts of normal physiology, dysregulated immune function and inflammation, whether accompanied by uterine dysbiosis or not, seem to trigger chronic uterine inflammation [7]. Early postpartum cows also undergo a period of hypocalcemia and increases in the concentrations of non-esterified fatty acids (**NEFA**) and blood β-hydroxybutyrate (**BHB**) in circulation from adipose mobilization. These changes may be adaptive or associated with an increased risk of disease or lessening production or fertility. In addition to hypocalcemia and elevated NEFA and BHB, elevated concentrations of the acute phase protein (**APP**) haptoglobin (**HP**) in circulation have been associated with an increased risk of metritis, endometritis, or purulent vaginal discharge (**PVD**) [8,9]. Excessive alterations in these blood markers in the early postpartum period may be indications of transition maladaptation.

Large observational studies have demonstrated repeatedly that cows in higher-risk categories based on blood markers or cows with uterine or non-uterine clinical diseases are more likely to have impaired reproductive function and performance [10,11,12,13]. However, the mechanisms linking these risk factors with reproductive dysfunction are not fully understood, particularly risk factors related to alterations in metabolic and inflammatory markers that are not always accompanied by a clinical manifestation of disease. It is plausible that metabolic imbalance and inflammatory disorders in the early postpartum period have short and long-term effects on different aspects of the reproductive system, resulting in prolonged anovulation, decreased estrus expression, decreased probability of pregnancy, and increased risk of pregnancy losses. Nutritional management strategies and treatment interventions, including hormonal synchronization protocols, are critical tools to improve transition health and fertility and may mitigate the negative consequences of health disorders on reproductive performance [14,15,16], but these topics are beyond the scope of this narrative literature review. Here, we describe the physiology of the transition period in high producing dairy cows that relates to health, such as lipid mobilization, hypocalcemia, systemic inflammation, and uterine disease. We discuss perspectives on the physiological mechanisms that may underlie the associations between transition health disorders and functions of the CL, follicle, oocyte, and uterine environment that in turn result in impaired reproductive performance.

## 2. Transition Health

### 2.1. Parturition and Transition Physiology

The period of transition from gestation to the onset of lactation, traditionally comprising 3 weeks before to 3 weeks after parturition, is characterized by intense physiological changes that often impose challenges to the maintenance of postpartum health, fertility, and productivity [17]. While mammary tissue expansion and milk synthesis start weeks before calving, the onset of parturition is preceded by endocrine changes, characterized by peaks in growth hormone, glucocorticoids, estrogen, and prolactin, and a decline in progesterone (**P4**) in circulation [18]. The maturation of the fetal hypothalamic–pituitary–adrenal axis elicits a peak in fetal cortisol release that triggers the upregulation of cyclooxygenase 2 that could induce prostaglandin F_2α_ (PGF_2α_) release in trophoblast cells, leading to luteolysis and a decline in maternal P4, myometrium activation and cervical dilation, and parturition [19]. The period around parturition is also characterized by an increase in markers of systemic inflammation [20,21]. Although this could indicate enhanced activity of the immune system, a small study with nulliparous Holstein cows demonstrated that the increase in circulating leukocyte count prepartum was accompanied by impaired neutrophil function in the week before and after parturition [22]. Furthermore, in healthy cows, Cai et al. [23] reported that neutrophil function increased before parturition but decreased immediately after parturition. Interestingly, among cows that developed metritis or mastitis postpartum, immune function was impaired before parturition [23]. This suggests that impaired immune function before parturition predisposes cows to postpartum disorders, or that peripartum immune dysfunction is linked to other physiological and metabolic stressors that are also associated with the development of postpartum diseases. Up to half of postpartum dairy cows are expected to experience at least one clinical or subclinical health disorder, which is a risk factor for reduced fertility [10,12,24]. The prevalence of diseases commonly reported in postpartum dairy cows is summarized in Table 1.

### 2.2. Lipid Mobilization

Nutrient requirements to support lactation often surpass the nutrient supply achieved through feed intake. With estimates from 1995, healthy dairy cows producing 30 kg of milk at 4 days in milk (**DIM**) require approximately 33 Mcal/d of net energy and 2.2 kg/d of metabolizable protein for milk synthesis and maintenance; however, they might be able to consume about 24 Mcal/d net energy and 1.7 kg/d metabolizable protein, representing only 73 and 77% of their nutritional needs [18]. At calving, the requirement of Ca for colostrum synthesis was estimated to be more than twice the requirement in late gestation [35]. Hence, it is not surprising that cows undergo marked nutritional deficits that can start before parturition and last until feed intake increases sufficiently to meet nutritional demands, days to six weeks or more postpartum.

Homeorhetic adaptations at the onset of lactation coordinate nutrient partitioning to support and prioritize milk synthesis while maintaining vital functions [36]. Some of these key metabolic adaptations include increased adipose tissue mobilization, reduced circulating insulin concentration and insulin sensitivity of adipose and skeletal tissues, increased hepatic gluconeogenesis, and consequently increased glucose uptake by the mammary gland [37]. As summarized by Drackley [17], the increased rate of lipolysis increases NEFA in circulation to be used as an energy source, but when excessive, NEFA is taken up by the liver. Hepatic NEFA metabolization occurs either through esterification into triglycerides and deposition in the liver, peroxisomal β-oxidation and complete mitochondrial oxidization into CO_2_, or incomplete mitochondrial oxidization to ketone bodies (e.g., BHB) which are released back into circulation. Excessive oxidization of NEFA is thought to have negative effects on feed intake, further exacerbating the mobilization of adipose tissue [38]. Although adipose tissue mobilization and the hepatic oxidation of fuels additional to glucose is likely an evolutionary adaptation to the onset of lactation, when excessive, it may predispose cows to health disorders.

Markers of the elevated mobilization of adipose tissues have been often associated with impaired health and fertility. Cows with elevated circulating NEFA (≥0.5 to 1.0 mM) or hyperketonemia (BHB ≥ 0.9 to 1.4 mM) in the first 2 weeks postpartum were at greater risk of displaced abomasum (**DA**) or metritis [28,39], endometritis or PVD [10,25], delayed onset of cyclicity [10], reduced estrus detection or pregnancy per AI [10,12,40], and increased risk of pregnancy loss [41]. Furthermore, herds with over 12 and 23% prevalence of hyperketonemia (BHB ≥ 1.4 mM measured once in the first 14 DIM) had decreased risk of pregnancy at first AI and increased risk of culling in the first 60 DIM, respectively [31]. The prevalence of cows in high-risk categories of circulating NEFA and BHB was presented in a separate review [16]. Nonetheless, the associations of elevated NEFA or hyperketonemia with subsequent health or reproduction are likely confounded by other health disorders. It is possible that the unfavorable associations of elevated NEFA or hyperketonemia with other disease processes are mediated through effects on immune function or other physiological stressors, such as hypocalcemia and systemic inflammation, as demonstrated in some, but not all, in vitro and in vivo studies [42]. Alternatively, alterations in these metabolic markers could be a consequence of the immune activation that begins as a part of physiological changes around parturition [4]. In this perspective, the rapidly increased glucose requirement for immune activation and the potential negative effects of immune activation on appetite would predispose cows to a higher degree of metabolic imbalance reflected in NEFA, hyperketonemia, or hypocalcemia, whereby these conditions are markers of immune activation but explicitly not causal of an increased risk of health disorders and infertility. It remains unclear if elevated NEFA, hyperketonemia, or dyscalcemia are simply markers of common antecedent physiologic risk factors or may, in combination with other variables, contribute to subsequent unfavorable outcomes. Experimental models to replicate these conditions and isolate their effects in the complex milieu of transition cows are elusive and will likely remain so.

### 2.3. Hypocalcemia

The rapid Ca demand for colostrum and milk synthesis often exceeds the homeostatic capacity to maintain eucalcemia, leading to a marked decline in circulating Ca between 1 and 4 DIM. Up to 5% of mature cows might develop milk fever, but subclinical hypocalcemia, traditionally based on a 2.0–2.2 mM cutpoint for total Ca in circulation, has been reported to affect up to 25% of primiparous and nearly 50% of multiparous cows [29,30]. The varying cutpoints and respective prevalences were summarized elsewhere [16]. Intracellular Ca signaling is involved in immune activation and function, which is impaired around parturition in association with a decline in circulating Ca [43]. In nonpregnant, nonlactating cows, induced subclinical hypocalcemia caused reduced neutrophil function, reduced rumen motility, decreased feed intake and circulating insulin, and increased NEFA [44]. Goff et al. [45] reported a decrease in rumination time from 1 d before to 3 d after parturition that was more pronounced in cows that had subclinical or clinical hypocalcemia. Likely consequently, hypocalcemia is associated with an increased risk of early lactation diseases, such as ketosis, retained placenta (**RP**), metritis, endometritis, or with decreased reproductive performance [9,46,47].

Most studies diagnosed hypocalcemia based on blood Ca measured at a single time point within 48 h postpartum. McArt and Neves [48] investigated health status and milk production in 407 cows with different dynamics of subclinical hypocalcemia in the first 4 DIM. In multiparous cows, the prevalence of transient hypocalcemia (blood Ca ≤ 1.77 at 1 DIM and >2.20 mM at 4 DIM), persistent hypocalcemia (blood Ca ≤ 1.77 at 1 DIM and ≤ 2.20 mM at 4 DIM), and delayed hypocalcemia (blood Ca > 1.77 at 1 DIM and ≤2.20 mM at 4 DIM) was 19, 13, and 27%, respectively. Multiparous cows with transient hypocalcemia were less likely to develop hyperketonemia (BHB ≥ 1.2 mM in the first 10 DIM), metritis, or DA, and had greater milk yield than cows with persistent or delayed hypocalcemia. Primiparous cows with persistent or delayed hypocalcemia (23 and 13% prevalence; based on 2.15 mM threshold for blood Ca at both 1 and 2 DIM) were more likely to have health disorders than normocalcemic cows, while those with transient hypocalcemia (17% prevalence) had greater milk yield than the other groups. The same research group has proposed a simplified classification of “dyscalcemia” (serum total Ca ≤ 2.20 mM at 4 DIM in multiparous cows), which is associated with lesser postpartum activity and rumination time [49]. Multiparous cows with persistent or delayed hypocalcemia also had reduced feed intake during the first 3 wk of lactation and decreased milk yield in the first 6 wk than normocalcemic cows or cows with transient hypocalcemia [50]. It is unclear what determines the pattern of postpartum hypocalcemia, but it could be linked to the extent of the immune activation and its implications for nutrient availability and feed intake [4]. For example, lipopolysaccharide (**LPS**)-induced acute systemic inflammation caused a 50% decrease in circulating ionized Ca in lactating cows [51]. Regardless, the resilience of transition cows to postpartum hypocalcemia, and the interaction between hypocalcemia and immune function are important factors for the risk of other postpartum health disorders.

### 2.4. Systemic Inflammation

Physiological changes and metabolic stress during the transition period are associated with changes in the immune system [14]. Cows undergo immune activation during the transition period that is likely a result of normal physiological changes in the mammary gland, uterus, and gastrointestinal tract [4,6]. During dry-off, the mammary gland undergoes rapid remodeling and involution, followed by a rapid proliferation and expansion of epithelial cells for colostrum synthesis and the onset of lactation. The activation of inflammation has been observed in association with mammary tissue remodeling and involution [52], and with cell turnover and function [53]. Other sources of immune activation and inflammation likely include uterine tissue damage and placental expulsion around parturition, uterine infection, and leaky gut due to increased gastrointestinal tract permeability attributed to diet changes or substantially reduced feed intake [4,6,7].

Parturition consists of a series of cyclooxygenase-mediated pro-inflammatory processes in the endometrium to promote uterine contraction and cervical dilation [19]. Following parturition, adequate immune function seems to be necessary for a spontaneous expelling of fetal membranes. The cows with RP had decreased circulating neutrophil function before and after calving compared to the cows that expelled the placenta spontaneously [54]. Furthermore, a small study observed reduced expression of genes associated with pro-inflammatory cytokine function (IL-1β, IL-6, IL-8, TNFα) in the utero-placental tissues of the cows that had RP compared to the cows that did not [55]. Regardless of RP or other calving-related disorders, uterine inflammation is expected to occur during the first week postpartum [56]. Uterine influx of immune cells and abundance of pro-inflammatory cytokines were observed at 7 DIM compared to 42 DIM in multiparous cows [57]. Furthermore, Cheong et al. [21] demonstrated that multiparous cows with more uterine inflammation at calving (≥35% polymorphonuclear cells in uterine flush) were more likely to ovulate the first dominant follicle postpartum than those with less uterine inflammation. It seems that adequate immune function and mild uterine inflammation in the first few days after calving are important to promote uterine tissue repair, placenta expulsion, and the reestablishment of ovarian function. It also suggests that inflammatory mechanisms in the uterus, at least to some extent, are linked to physiological processes that might be necessary for a successful transition.

Besides tissue trauma, inflammation is mainly triggered by an activation of the innate immune system in response to stimuli by pathogens. In peripheral tissue, pathogen-associated molecular patterns are recognized by cellular pattern recognition receptors, initiating a cascade of events intracellularly that results in the upregulation and release of pro-inflammatory cytokines systemically, such as IL-1β, IL-6, and TNFα [58]. Besides the recruitment of leukocytes and macrophages, pro-inflammatory cytokines trigger the production and secretion of acute-phase proteins in hepatocytes, including HP, serum amyloid A, and LPS-binding protein [59]. Inflammatory processes also result in greater production of reactive oxygen species (**ROS**), which are unstable free radical molecules that can cause damage to DNA, RNA, lipids, and proteins, and have been shown to affect reproductive tract tissues [60]. Inflammatory processes can trigger local or systemic inflammation, as an increase in blood HP concentration immediately after parturition has been observed in clinically healthy cows [56,61]. A growing number of studies have demonstrated circulating HP concentration in the first week postpartum as a potentially useful marker of predisposition to subsequent disease and reproductive problems [16]. A more pronounced increase in blood HP occurred in cows that subsequently developed metritis [20], clinical or subclinical endometritis [9], or in cows that failed to ovulate the first dominant follicle [21]. It was also associated with reduced estrus detection and pregnancy rate or increased days open [12,62,63]. It seems that some degree of systems and uterine inflammation around parturition is a physiological process, but dysregulated inflammation (likely accompanied by infectious processes, e.g., in the uterus) might trigger systemic inflammation or chronic uterine inflammation [7], further predisposing cows to other health disorders. However, our recent experiment using intrauterine LPS challenge did not support the hypothesis that uterine inflammation would cause systemic inflammation, at least under the conditions tested [56].

Dietary changes during the transition period may also contribute to some degree of systemic inflammation. Alterations in feeding behaviour and increased dietary starch content can alter gut microbiota and reduce rumen pH, potentially damaging the epithelial barrier and promoting LPS translocation to portal circulation through “leaky gut” [64]. Studies have demonstrated an increase in circulating concentrations of APP after feeding primiparous cows with high-grain diets (30 to 45% DM compared to 0 to 15% DM) [65] or after inducing a reduction in intestinal barrier function [66]. These reports provide evidence that LPS can translocate systemically from the gut due to dietary changes or intestinal barrier dysfunction and induce systemic inflammation and increase susceptibility to disease.

Inflammation may also be triggered by the mobilization of adipose tissue to support lactational energy requirements. Lipolysis normally occurs through a classic lipolytic pathway in which hormone-sensitive lipase is activated by protein kinase A, but it may occur through an inflammatory pathway where it is activated through mitogen-activated protein kinase/extracellular signal-regulated kinase signaling and Toll-like receptor (**TLR**) 4 [67]. Both classic and inflammatory lipolysis pathways can be activated by LPS, which can also reduce insulin sensitivity in adipose tissue [67]. From 10 d before to 21 d after parturition, cows had infiltration of macrophages in the adipose tissue, which was exacerbated in cows losing ≥ 0.5 point (1 to 5 scale) of body condition score (**BCS**) [68]. In humans, the resulting increase in circulating fatty acids from lipolysis, including arachidonic, palmitoleic, and saturated fatty acids, has been associated with a pro-inflammatory state [69]. Some of the suggested pathways linking circulating fatty acids and inflammation are through the modulation of adipokine secretion, promotion of a pro-inflammatory cascade by binding to TLR, or alteration of cellular (such as macrophage) function through the activation of peroxisome proliferator-activated receptors [69]. In obese humans, chronic low-grade systemic inflammation is observed in association with increased circulating fatty acids and insulin resistance [70]. In transition cows, a pro-inflammatory state associated with elevated BCS can be observed as early as 2 weeks prepartum: Bogado Pascottini et al. [71] compared the lipid and hepatic transcriptome profiles of multiparous cows with high (≥4) vs. intermediate (2.75 to 3.50; 1 to 5 scale) BCS 2 weeks before expected parturition. Besides greater adipose tissue weight, over-conditioned cows had greater serum NEFA concentrations and liver lipid content, and upregulation of the genes associated with pro-inflammatory acute-phase response. Excessive and dysregulated adipose tissue mobilization may be both triggered by and contribute to a pro-inflammatory state, increasing the susceptibility of cows to health disorders.

Systemic inflammation in transition cows, regardless of the occurrence of clinical disease, may interfere with the neuroendocrine systems regulating feed intake [72]. It has been speculated that saturated fatty acids, pro-inflammatory cytokines, and LPS in circulation can be sensed by hypothalamic neurons, eliciting an inflammatory response in hypothalamic microglia cells, and impairing endocrine functions that control feed intake. That aligns with the 24% reduction in feed intake observed in cows administered bovine recombinant tumor-necrosis factor in the first week postpartum [73]. The reduction in feed intake may further exacerbate negative energy balance, lipolysis, and inflammation. Other sources of systemic inflammation are common early postpartum infectious processes, such as mastitis and metritis. From intramammary or uterine infections, endotoxins released into circulation can elicit an inflammatory response locally and systemically. The activity of immune cells clears pathogens, but can also damage mammary epithelial and secretory cells, reducing milk synthesis [74]. In the uterus, inflammation is essential to promote pathogen clearance and tissue repair after parturition as previously discussed, but in some cases, it can develop into systemic illness or chronic uterine inflammation.

Current thinking is that almost all cows undergo negative energy balance and adipose mobilization, as well as immune activation and low-grade, systemic inflammation during the transition period that is not necessarily accompanied by a metabolic or clinical disease. However, the stimuli for systemic inflammation and excessive mobilization of body fat are likely multifactorial and still not fully understood. We recently conducted a study testing the hypothesis that systemic and uterine inflammation can be triggered reciprocally in clinically healthy cows using intravenous or intrauterine LPS challenge models at 5 or 40 DIM [56]. We expected differences in immune dysfunction, metabolic stress [75], and systemic and uterine inflammation [5,6] at 5 vs. 40 DIM. At 5 DIM, systemic LPS challenge resulted in subtle and inconsistent alterations in markers of systemic inflammation, likely due to a high variation in baseline inflammatory status of cows that are exposed to endotoxins naturally following parturition. At 5 DIM, uterine LPS did not result in greater uterine inflammation. On the other hand, at 40 DIM there was a substantial increase in markers of systemic inflammation following intravenous LPS, and an increase in endometrial PMN following intrauterine LPS. It is still unclear whether uterine inflammation contributes to systemic inflammation (or vice versa) in clinically healthy cows. A better understanding of the causes and effects of metabolic and inflammatory disorders, whether in association with endotoxins or other mechanisms, will clarify the determinants and potential mitigation strategies for impaired health and fertility associated with excessive or persistent inflammation.

### 2.5. Uterine Disease

Almost all cows are expected to have bacterial contamination of the uterus immediately after calving. However, uterine disease can develop when there is insufficient resilience of cows in avoiding, tolerating, or resisting pathogenic bacteria, such as failure to maintain optimal immune function and epithelial tissue integrity, neutralization of toxins, and tissue repair [76]. Dysregulated immune function may also result in RP [55] and in exacerbated inflammation in response to endometrial tissue damage by bacterial toxins [77]. This can increase susceptibility to acute or chronic reproductive tract diseases such as metritis, PVD, and endometritis [78]. Metritis, characterized clinically by fetid discharge and histologically by inflammation in all uterine tissues in association with dysbiosis of the uterine microbiota [79], is diagnosed in 13 to 20% of cows in the first 2 to 3 weeks postpartum [12,25,78]. Risk factors for metritis include decreased feed intake pre- and postpartum [80], elevated concentrations of NEFA (≥0.6 mM) in blood one week before parturition, elevated HP in the first (≥0.8 to 1.0 g/L) or second week postpartum (≥0.4 g/L) but preceding metritis, hypocalcemia (≤2.15 mM) in the first 3 DIM, dystocia, or RP [20,25,46]. Cows with elevated blood HP or metritis are at greater risk of chronic uterine diseases such as PVD and endometritis (defined as ≥6% polymorphonuclear cells in endometrial cytology), which affect approximately 15% and 20% of cows in the second month of lactation, respectively [12,25]. However, the prevalence of PVD and endometritis can vary among herds from approximately 5% to 40% each [33]. Longer-term consequences associated with elevated HP in circulation, metritis, endometritis, or PVD include delayed resumption of ovulation and decreased estrus detection and reproductive performance [10,12,41,81].

## 3. Mechanisms Linking Disease and Reproductive Function

### 3.1. Dominant Follicle Function, Estrus, and Ovulation

Mechanisms underlying the associations between health disorders and reproductive dysfunction are likely numerous and not fully understood. One likely pathway in the early postpartum cow is through impaired hypothalamic–pituitary–ovarian axis function. Hypothalamic kisspeptin neurons are key regulators of GnRH and subsequent gonadotrophin secretion, and kisspeptin neurons can be suppressed by LPS and or inflammatory mediators [82]. This would result in alterations in gonadotrophin release, altering follicular growth and maturation and preventing or delaying ovulation. Monteiro et al. [83] evaluated the characteristics of follicles in anovular cows diagnosed with health disorders. They speculated that, in 268 anovular cows examined at 35 and 49 DIM, the size of the largest follicle would indicate whether cows had impaired FSH pulses, LH pulses, or a lack of an LH surge. A follicle ≤ 7 mm in diameter would indicate FSH pulse deficiency, whereas a follicle of 8 to 13 mm or 14 to 17 mm in diameter would indicate low and intermediate LH pulsatility, respectively. A follicle ≥ 18 mm would indicate ovulation failure due to lack of an LH surge. Compared to anovular cows with the largest follicle ≥ 18 mm (32% of the anovular cows), those with the largest follicle being 8 to 13 mm (30% of the anovular cows) had a greater number of disease events (RP, metritis, hyperketonemia, mastitis, lameness, respiratory, or digestive problems), higher somatic cell count in milk at 30 DIM, lower BCS at 35 DIM, and lower milk yield between 35 and 49 DIM. Most of these variables were not different between anovular cows with the largest follicle ≥ 18 mm and cyclic cows. This implies that the effects of health disorders on anovulation possibly occurred by impairing follicular maturation from dominance to preovulatory size and ovulation when follicles are dependent on LH pulsatility and LH surge, respectively.

The direct or indirect effects of postpartum metabolic or inflammatory disorders on follicular development have been discussed in detail [60,82,84]. As follicles take 60 d or more to grow from the early preantral stage to the preovulatory stage, follicles expected to ovulate during the breeding period were in their early stages of growth during the first few weeks postpartum. The early development of follicles and involution and the remodeling of the uterus postpartum occur concurrently with metabolic alterations such as high rates of lipolysis from BCS loss, elevated blood NEFA and BHB, and high liver triacylglycerol, and these can impair reproductive processes both before and after the first ovulation [14]. In addition to metabolic alterations, reproductive function can be disrupted by inflammatory processes in the reproductive, particularly if accompanied by infection [60]. During uterine infection, LPS, microbial byproducts, and inflammatory mediators can translocate systemically and locally to the ovaries [60], and granulosa cells from emerging follicles can sense endotoxins, triggering local inflammation [85]. In cows with high uterine bacterial contamination (above the 75% percentile of bacterial score) examined from 7 to 28 d, the dominant follicle (>7 mm) was smaller in the first follicular wave and the circulating concentration of estradiol (**E2**) was lesser compared to cows with less bacterial contamination [86]. In heifers, infusion of LPS into the uterine lumen after induced luteolysis suppressed the preovulatory LH surge and ovulation [87]. In follicular fluid from cows diagnosed with endometritis 40 to 60 DIM, LPS concentrations were greater compared to healthy cows, and follicles ≥ 4 mm produced less E2 and P4 in granulosa cells when exposed to LPS in vitro [88]. Similar effects were seen in an in vivo study measuring concentrations of LPS in follicular fluid and steroidogenesis by theca and granulosa cells of ovaries collected from multiparous cows in an abattoir [89]. Follicles with greater LPS concentrations had lower concentrations of E2 and lower expression of LH and FSH receptors than follicles with lower LPS concentrations. It seems that LPS and the oxidative damage caused by ROS during uterine infection and inflammation can affect the function of follicles and contribute to infertility.

Several studies have examined the effects of immune activation and systemic inflammation on reproductive endocrinology by challenging cows with LPS systemically or in other tissues [82]. In ewes, administration of intravenous LPS stimulated acute systemic inflammation with heightened expression of IL-1β, IL-6, and TNFα genes and their receptors, and inhibited expression of GnRH and LH receptors, in the anterior pituitary 7 d after administration [90]. Similar LPS infusions in ewes reduced the amplitude of GnRH and LH pulses and the frequency of LH pulses in hypophyseal portal blood 10 h after infusion [91]. The systemic LPS challenge caused a slower increase in E2 during follicular growth and caused a delayed (or lack of) estrus and LH surge in comparison to controls [91]. Interestingly, three of the LPS-challenged ewes had abnormal LH pulse patterns, while the remaining three had LH pulse patterns similar to controls even though the E2 rise and LH surge were impaired. In a study with 27 Holstein cows, Lavon et al. [92] induced intramammary infections and synchronized cows to have four subsequent 7-d estrous cycles. Those receiving LPS to induce mastitis had lower concentrations of E2, androstenedione, and P4 in follicular fluid in the cycle immediately after treatment compared to untreated cows, with concentrations returning to normal in the third and fourth cycles. Interestingly, cows receiving *Staphyloccos aureus* exosecretions (extract from *S. aureus* to induce subclinical mastitis) had a smaller number of follicles and tended to have lower follicular fluid E2 and androstenedione concentrations only in the third and fourth cycles compared to untreated cows. In this study, however, the induced estrous cycles were shortened and evaluated over a 28-d period only, so it is unclear if longer term effects would exist in spontaneous estrous cycles. Similarly, an experiment testing the effects of intramammary or intravenous LPS administration during synchronized estrus in mid-lactation Holstein cows [87] observed one-third of LPS-treated cows having delayed ovulation after estrus, with a lesser or delayed LH surge compared to controls. Only a few cows treated with intravenous LPS had a second E2 peak and standing estrus prior to delayed ovulation. These studies suggest that a lack of estrus expression and ovulation failure is likely a result of impaired maturation and function of the dominant follicle. Under natural conditions, this could be caused by endotoxins from the reproductive tract or other tissues eliciting systemic inflammation and altering the reproductive endocrine system.

### 3.2. Inflammation and Luteal Function

Physiological processes within the ovary, such as the formation and regression of the CL, involve the action of inflammatory mediators. Ovulation involves tissue damage through rupture of the dominant follicle. The LH surge preceding ovulation induces uterine epithelial cells to release prostaglandin E_2_ (**PGE**), which acts to reduce the phagocytic activity of neutrophils towards sperm cells in the uterine tube [93]. During the luteal phase, PGE has antiluteolytic and luteotropic effects and may be involved in the maintenance of the CL [94]. Following ovulation, the early CL produces chemoattractant IL-8 that promotes the migration of neutrophils to the CL, participating in the regulation of angiogenesis for CL development [95]. During the late luteal phase in the absence of an embryo, pro-inflammatory mediators such as IL-1β, TNFα, and IFN-γ are involved in the stimulation of PGF_2α_ release by endometrial cells for luteolysis [96]. If an embryo is present, IFN-τ secreted by the trophoblast inhibits oxytocin receptors in the endometrium and prevents luteolytic pulses of PGF_2α_ to maintain pregnancy [97].

Because inflammatory mediators participate in normal CL physiology, it is plausible that infectious and inflammatory processes in the uterus and ovaries may disrupt CL function. In an observational study, we recently evaluated the spontaneous P4 profiles from 35 to 70 DIM in postpartum cows that had indications of both systemic inflammation in the first week postpartum (serum HP ≥ 0.8 g/L) and endometritis (≥6% endometrial PMN) at 35 DIM compared to healthy cows [98]. Cows with inflammatory disorders had a longer time to first ovulation and were more likely to have prolonged luteal phases (>21 d) than healthy cows. In an LPS challenge administered systemically after induced luteolysis in heifers, LH pulse frequency was decreased during follicular growth, E2 production by the dominant follicle was decreased, and the LH surge was delayed [99]. Consequently, heifers administered LPS had delayed CL formation and a delayed rise in circulating P4 following ovulation. Similarly, lactating Holstein cows had a delay in LH surge and ovulation after administration of intravenous or intramammary endotoxin immediately after detected estrus [100]. During the luteal phase in nonlactating, clinically healthy Holstein cows, Herzog et al. [101] infused LPS systemically 10 d after ovulation and reported a decrease in CL size through the rest of the luteal phase, a decrease in luteal blood flow for 72 h after treatment, and a decline in circulating P4 compared to control cows. Interestingly, LPS infusion increased plasma PGF_2α_ tenfold within 30 min after administration and increased plasma PGE twofold 1 h after infusion. Similarly, the induced subclinical endometritis by repeated intrauterine LPS infusion from 12 h before to 9 d after ovulation reduced CL size and blood flow, decreased circulating P4 concentration, increased PGF_2α_, and induced premature luteolysis in heifers [102]. These alterations were accompanied by increased luteal expression of PGE synthase and apoptosis-related factor caspase-3. In an observational study [103], 14 cows with pathogenic bacteria isolated from the uterus in the first 15 DIM and either PVD or fetid discharge (i.e., metritis) were examined twice weekly until 35 to 45 DIM. Besides the greater concentrations of endotoxin in uterine fluid and plasma, the cows with metritis had greater concentrations of PGE in the uterine fluid and PGF_2α_ metabolite (PGFM) in plasma in the first two weeks than the cows with PVD. These findings suggest that circulating endotoxins could disrupt luteal function either by inducing luteolysis (through increased PGF_2α_) or by delaying luteolysis (possibly through the enhanced antiluteolytic activity of PGE). Therefore, it is likely that inflammatory disorders in postpartum cows partially explain unexpected estrous cycle patterns such as prolonged anestrous, lack of estrus detection by activity monitors in cycling cows, or abnormal luteal phases (prolonged luteal phases and suboptimal P4 concentrations); all associated with reduced fertility [16,104].

### 3.3. Effects of Endotoxins on Oocyte Competence or Uterine Environment

The ovary contains a variety of immune cells in the stroma as part of innate defense mechanisms that protect pre-antral follicles. This was demonstrated when, in response to ex vivo LPS, there was an accumulation of inflammatory mediators in supernatants of the ovarian cortex [105] and reduced E2 secretion [88]. The inflammation induced by LPS in ovarian tissues was linked to the increased activation of primordial follicles and consequently a reduced primordial follicle pool in bovine ovaries ex vivo and in mice in vivo [105]. As primordial follicles can take up to 180 d to become a preovulatory follicle [106], the early activation of primordial follicles induced by endotoxemia could affect the oocyte and mediate the long-term effects of inflammatory diseases on fertility.

Naturally occurring intramammary infections or ex vivo LPS have been suggested to affect oocyte maturation and function and the subsequent embryo development. In 50 Holstein cows, those with low milk somatic cell count (<200,000 cell/mL) before slaughter had a similar number of oocytes recovered at slaughter, but a greater proportion of in vitro-produced embryos developed to the blastocyst stage than from the cows with medium (200,000–600,000 cell/mL) or high somatic cell count (>600,000 cell/mL) (19% vs. 6% and 3%, respectively) [107]. This may mirror inflammatory mediators or LPS from naturally occurring intramammary inflammation and infection that translocate systemically and affect oocyte function. In vitro, increased concentrations of LPS disrupted the expansion of the cumulus–oocyte complex during ovulation and decreased the meiotic competence of bovine oocytes [108]. Similarly, oocytes exposed to LPS in vitro had lesser meiotic progression, mitochondrial membrane potential, and ability to develop to the blastocyst stage [109]. Exposure to LPS also reduced the survival of bovine embryos [110] and the number of trophoblast cells in bovine blastocysts [109]. These data demonstrate the inflammatory disorders, even occurring outside of the reproductive tract, can have immediate effects on oocyte function and long-lasting impacts on fertilization and embryo survival.

Endotoxins and the associated inflammatory responses can also have direct effects on the uterus. In vitro LPS exposure was associated with increased expression of several proteins in bovine endometrial epithelial cells associated with cell metabolism, apoptosis, and oxidative stress changes identified through proteomics [111]. These changes in protein profile could be linked to an impaired uterine function and early conceptus development and maintenance. The lipids accumulated in endometrial epithelial cells during diestrus are important sources of fatty acids for elongation of the preimplantation conceptus [112]. It was speculated that exacerbated mobilization of adipose tissues, common in early postpartum cows, increases concentrations of saturated and monounsaturated fatty acids in circulation during the same period. These fatty acids could be incorporated by the endometrium, altering the lipid composition and impairing cell metabolism and function in the early conceptus [113]. In studies evaluating uterine gland development and uterine morphology through histology and immunohistochemistry, cows diagnosed with metritis within 10 DIM or with PVD at 30 DIM had less glandular development at 30 DIM and increased pathological fibrogenesis, leading to endometrial fibrosis and adenomyosis (abnormal endometrial invasion into the myometrium) later postpartum, compared to healthy cows [114,115]. Together, these studies suggest that altered lipid metabolism due to excessive mobilization of body reserves and uterine inflammation, both common in the early postpartum period, can impair uterine environment, function, and morphology, and contribute to impaired fertilization and embryo development.

The studies discussed above mainly explored the negative effects of inflammation, generally with induced exposure to LPS, on the function of the uterus, follicles, CL, or oocytes. For follicular function and ovulation, it seems that the effects of inflammation can occur either indirectly through compromised hypothalamic–pituitary–ovarian axis function or directly through impaired follicular function. For instance, suppressed LH pulsatility could limit the maturation of a dominant follicle, while impaired follicular LH receptor expression could reduce its ability to respond to LH pulses and surge. If LH pulses and follicular maturation are compromised, E2 production will be reduced, requiring the follicle to produce E2 for longer until it reaches a critical concentration, delaying or inhibiting the LH surge and ovulation. For CL function, endotoxins or inflammatory mediators could either induce premature luteolysis (through increased PGF_2α_) or prevent or delay luteolysis (through enhanced antiluteolytic activity of PGE). Oocyte competence could be affected directly by endotoxins in the ovaries, or indirectly by premature activation of primordial follicles. Furthermore, intense metabolic alterations in the early postpartum period could result in a compromised uterine environment that impairs conceptus development. However, most studies used in vitro culture or models with induced endotoxemia with an immediate acute inflammatory response. It is possible that these models do not fully represent the varying patterns (grade and duration) of exposure to endotoxins, or of unrelated systemic inflammation that postpartum cows experience in the transition period. It is unclear if alterations in reproductive function are caused directly by endotoxins, by the resulting inflammatory response, or both. Potential strategies to mitigate the negative consequences of transition maladaptation on reproductive function, such as minimizing metabolic stress, supplementing micronutrients, or strategically using non-steroid anti-inflammatories or immune-enhancing agents, have been discussed [14], but require further investigation. Comprehensive studies that mimic naturally occurring disorders and metabolic alterations that are common in transition dairy cows are warranted to better understand the mechanisms linking metabolic and inflammatory disorders in the reproductive–endocrine system.

## 4. Conclusions

**Figure 1:**
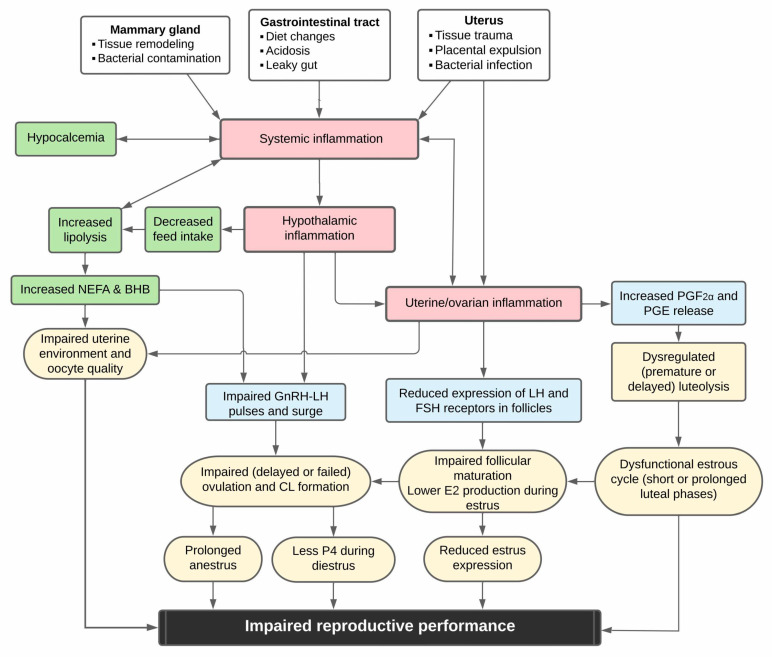
Schematic representation of potential links of transition health disorders with impaired reproductive function and fertility in dairy cows.

In dairy cows, the transition period is characterized by profound physiological changes, metabolic adaptations, and inflammatory processes. Health disorders occurring during the first days or weeks after parturition, characterized based on alterations in blood markers of metabolic adaptation or inflammation, or clinical disease, have negative carryover effects on reproductive function and performance for weeks to months. The findings from in vivo and ex vivo studies imply that this can occur due to the effects of metabolic and inflammatory disturbances on different aspects of reproductive function. This leads to impairments in dominant follicle function, CL, oocyte, and uterine environment. These may result in prolonged anovulation, reduced estrus expression or responsiveness to synchronization protocols, decreased P4 concentrations during diestrus, and impaired fertilization and pregnancy maintenance, and we describe a possible causal diagram in Figure 1. Systemic inflammation in the first week postpartum, assessed by changes in circulating concentrations of acute phase proteins seems to influence health and reproduction, but the optimal markers, levels, or durations that identify maladaptation or elevated risk of unfavorable outcomes remain to be characterized. It seems that the associations of metabolic disorders (i.e., dyscalcemia, elevated NEFA, or hyperketonemia) with health and reproductive outcomes are confounded by immune activation and inflammation. More research is needed into temporal and perhaps causal relationships among these. Future studies should attempt to better characterize the timing and extent to which inflammatory and metabolic disorders during the transition period are indications of transition maladaptation. With further advancements in this area, new strategies to monitor, prevent, or mitigate the impacts of transition maladaptation on reproductive function and performance can be developed to improve the health and fertility of dairy cows.

## Figures and Tables

**Table 1:** Incidence or prevalence of clinical health disorders commonly reported in early postpartum dairy cows.

Health Disorder	Definition	Incidence or Prevalence	References
Calving problems	Any of dystocia, twin birth, stillbirth, or retained placenta	9 to 15%	[10,12,25,26,27]
Clinical hypocalcemia	Farm personnel/veterinarian diagnosis or recumbent cow with serum total Ca < 2.0 mM	1 to 5%	[12,27,28,29,30]
Retained placenta	Failure to expel fetal membranes within 24 h after calving	5 to 12%	[12,25,27,28,31,32]
Clinical ketosis	Reduced feed intake or reduced milk yield with hyperketonemia (≥1.2 mM blood BHB)	3 to 10%	[26,27,28]
Displaced abomasum	Veterinary diagnosis of the left- or right-side displacement of abomasum within 30 or 60 DIM	2 to 4%	[12,27,28,31,32]
Metritis	Watery fetid vaginal discharge with or without systemic illness within 15 DIM	5 to 19%	[12,25,26,27,28,32]
PVD	≥50% pus in vaginal discharge at 35 to 56 DIM	10 to 25%	[12,25,32,33]
Clinical endometritis	≥5% PMN in endometrial cytology at 35 or ≥4% PMN at 56 DIM with PVD	4 to 7%	[25,32]
Subclinical endometritis	≥5% PMN in endometrial cytology at 35 DIM, ≥2% at 41 DIM, or ≥4% at 56 DIM without PVD	10 to 27%	[25,32,33]
Mastitis	Abnormal milk or visible inflammation in one or more quarters within 30 DIM, often based on treatment records only	3 to 15%	[10,12,26,28,32]
Lameness	Walking with altered gait or arched back, or defined arbitrarily by farm personnel diagnosis within 30 DIM	3 to 14%	[10,12,26,28]
Respiratory disease	Diagnosis of respiratory disease or pneumonia. Not commonly reported	2%	[27]
At least one clinical disease	Any of dystocia, RP, metritis, PVD, mastitis, lameness, ketosis, DA, or pneumonia	44 to 48%	[10,12,26,34]
Multiple clinical diseases	>1 of dystocia, RP, metritis, PVD, mastitis, lameness, ketosis, DA, or pneumonia	17 to 26%	[10,12,26]
Uterine disease	RP, metritis, endometritis, or PVD	22 to 45%	[10,12,34]
Non-uterine disease	Clinical hypocalcemia, clinical mastitis, lameness, or digestive or respiratory problems	23 to 32%	[10,12,34]

Abbreviations: DIM = days in milk; PMN = polymorphonuclear cells; PVD = purulent vaginal discharge; RP = retained placenta.

## Data Availability

No new data were created or analyzed in this study.
